# Advanced Self-Healing Ceramics with Controlled Degradation and Repair by Chemical Reaction

**DOI:** 10.3390/ma16196368

**Published:** 2023-09-23

**Authors:** Nobuhide Sekine, Wataru Nakao

**Affiliations:** 1 Graduate School of Engineering, Yokohama National University, Tokiwadai 79-5, Hodogaya-ku, Yokohama 240-8501, Kanagawa, Japan; 2 Faculty of Engineering, Yokohama National University, Tokiwadai 79-5, Hodogaya-ku, Yokohama 240-8501, Kanagawa, Japan

**Keywords:** smart materials, self-healing ceramics, corrosion, material design

## Abstract

Controlling the chemical reaction rate concerning degradation and repair is found to be important to design advanced self-healing ceramics. The recovery and degradation behaviors of strength and stiffness were investigated by exposing aqueous solutions of different pH and calcium ion concentrations to the introduced crack on typical self-healing ceramics dispersed with alumina cement as a self-healing agent. The chemical reaction of cement undergoes the following three stages: dissolution of components such as calcium ions, formation of a gel, and formation of final products. Experimental and thermodynamic assessments revealed that even under conditions where the final products are identical (thermodynamic equilibrium), kinetic effects (excessive dissolution of components or insufficient crystal formation) result in strength degradation rather than repair. It was also suggested that the repair function could be enhanced by controlling the nucleation site of the crystals.

## 1. Introduction

Recently, materials that utilize chemical reactions as a healing (repair) function have been developed and studied. Conventional self-healing ceramics utilize oxidation reactions of non-oxides as the healing function and can only be used at temperatures above 800 °C [1,2,3,4,5,6]. To further expand the range of applications, lowering the temperature at which healing occurs is investigated.

For self-healing materials to regain their strength, it is essential to go through the following three stages: the inflammation stage, in which the reaction begins; the repair stage, in which fluid materials fill the cracks; and the modification stage, in which the filled materials solidify and regain strength [7,8,9,10,11,12,13,14,15]. In particular, crack filling by the formation of flowable material during the repair stage is important for complete strength recovery. For example, in SiC/Al_2_O_3_ self-healing ceramics, it is known that a supercooled melt forms during the reaction process, which fills cracks and shows complete strength recovery [7]. Conversely, although crack healing behavior was observed in Co/Al_2_O_3_ [16] and Fe/Y_2_O_3_/mullite [17], complete strength recovery does not occur because there is no repair stage. Also, self-healing ceramics that do not require flowable materials and have different healing mechanisms have been reported. In these ceramics, the outward diffusion of cations takes the place of flowable materials by filling cracks; examples include Ni/Al_2_O_3_ [18] and self-healing ceramics utilizing the MAX phase [19,20,21,22,23]. The self-healing function utilizing outward diffusion can be utilized in relatively high-temperature atmospheres where diffusion in the solid phase is active.

To decrease the temperature at which the self-healing function occurs, it is more reasonable to promote the formation of fluid materials at low temperatures than with diffusion, which requires high temperatures. Recently, in a SiC-Al_2_O_3_-B_4_C ceramic composite, liquid-phase B_2_O_3_ generated from B_4_C successfully recovered strength at 700 °C for 30 min by filling cracks [24]. For self-healing ceramics that exhibit healing functions at even lower temperatures, Sekino et al. reported a Ti/Al_2_O_3_ composite material [25]. Ti/Al_2_O_3_ has succeeded in self-healing at room temperature by utilizing the anodic oxidation of Ti as a healing function. However, complete self-healing (100% strength recovery and crack filling) does not occur because there is no formation of flowable material during the repair stage. There are few examples of ceramics that are completely self-healing at room temperature, and the current state of knowledge is insufficient to create room-temperature self-healing ceramics.

The effect of temperature is expected to be significant during the repair stage as well as the inflammation and modification stages. At high temperatures, the reaction rates of chemical reactions are generally fast, and the reaction rates of forward as well as reverse reactions are extremely fast. Therefore, thermodynamic equilibrium is easily achieved, and the final product tends to be a homogeneous and dense material. However, in chemical reactions at low temperatures such as room temperature, the reaction rate of chemical reactions is generally slow, reverse reactions are difficult to occur, and diffusion of materials takes time. Therefore, thermodynamic equilibrium is difficult to achieve at low temperatures, and the ever-changing reaction field tends to make the final products heterogeneous and weak.

In addition to repair, the effects of degradation associated with the reaction should also be considered. For example, conventional self-healing ceramics use the oxidation reaction of SiC for their self-healing function, and it is known that CO gas is generated and emitted during oxidation (from the inflammation stage to the repair stage). In the case of SiC, this is not a particular problem because the effect of degradation is small. However, the effect of such degradation is expected to be large for reactions with relatively high reaction rates from the inflammation stage to the repair stage. Thus, in the inflammation, repair, and modification stages, degradation and repair compete kinetically with each other. For these reasons, to develop self-healing ceramics with high healing abilities at room temperature, it is necessary to clarify the kinetic effects of degradation and repair in the elementary reaction process on the healing ability at room temperature.

In this study, alumina cement was dispersed in a ceramic matrix to develop ceramics with room-temperature self-healing ability. Cement is a typical self-healing material at room temperature [26,27,28,29] and this was applied to ceramics. Alumina cement is composed mainly of calcium, and the CaO component leaches out when exposed to water. When the atmosphere becomes alkaline, the Al_2_O_3_ component begins to leach out and Al(OH)^4−^ is formed; Al(OH)^4−^ forms a sol, which reacts with Ca^2+^ to form several kinds of hydrates [30,31]. In this study, the kinetic influence of the reaction process on the healing function was investigated by comparing thermodynamic calculations and experiments.

## 2. Materials and Methods

A powder mixture of sand, which is generally used for ceramics(LIXILCorporation, Tokyo, Japan), and alumina cement (Denka High Alumina Cement Super, Denka Company Limited, Tokyo, Japan) with a volume ratio of 30% was prepared, compacted uniaxially at 25 MPa, and then the temperatures were increased at 5 °C/min in an electric furnace(NITTO KAGAKU CO., Ltd., Nagoya, Japan), held at 1150 °C for 10 h, and cooled at 5 °C/min to produce a sample with room-temperature self-healing function. The chemical compositions of raw materials are shown in Table 1. After the sintering, the material is mainly composed of CaAl_2_Si_2_O_8_, Al_2_O_3_ (corundum).

Strength recovery was confirmed by comparison of three-point bending tests before and after healing. The specific method is described below. Three-point bending test specimens were prepared according to JISR 1601, and pre-cracks were introduced by indentation of 2 kg using a Vickers test machine(Matsuzawa Co., Ltd., Akita, Japan). The specimens were then subjected to a “healing process” under the conditions shown in Table 2. For the “healing process,” a pH-adjusted solution was dropped onto the pre-cracked specimens in a container with 100% humidity as shown in Table 2, and the drops were kept for 12 h, with the drops repeated as necessary to prevent drying out. It is important to note that this method was used in this study to minimize as much as possible the loss of components in early stages of the reaction and to focus on the effect of the reaction rate. The pH values were 9.4, 11.4, and 12.4, respectively. Calcium hydroxide was used for the preparation of pH 9.4 and pH 12.4, and ammonium solution for the preparation of pH 11.4. The specimens were then dried in an incubator(AS ONE CORPORATION, Osaka, Japan) at a controlled temperature and humidity for 72 h. The temperature and humidity were adjusted to 25 °C and 0%, respectively, by placing silica gel in the incubator. Then, the specimens were subjected to a three-point bending test(SHIMADZU CORPORATION, Kyoto, Japan). The span and head speed were 30 mm and 0.5 mm/min, respectively.

The thermodynamic calculation software FactSage 8.0 was used to calculate the phases when in equilibrium with the aqueous solution under the conditions shown in Table 2. The database was created by referring to the cement thermodynamic data Cemdata18 [32] and used in the calculations.

## 3. Results and Discussion

**Figure 1:**
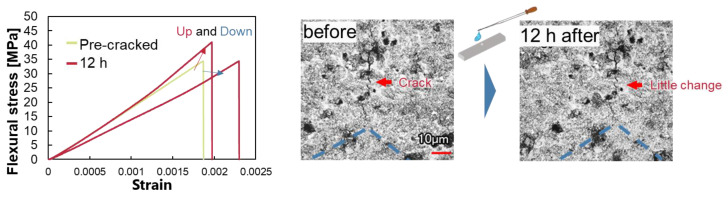
Healing behavior under pH 9.4 and [Ca^2+^] = 2.29 × 10^−7^.

Figure 1 shows the healing behavior for the case of healing with calcium hydroxide at pH 9.4. The green line showed in the figure is the pre-cracked material, and the red line is the one treated with the aqueous solution for 12 h. Three specimens were taken for each condition, and only representative data are shown. Under this condition, there was an increase in strength and stiffness in some cases and a decrease in others. No significant changes in the cracks were observed when comparing the surface cracks before and after 12 h of healing even though the strength and stiffness changed. This can be explained by the fluctuation of chemical reactions. In this condition, the concentration of the chemical species in water was too low to proceed with the chemical reaction after dissolution, so degradation caused by dissolution of the chemical component from the crack walls was dominant. However, in some cases, the concentration of chemical species came to be locally high inside the crack and precipitation could occur at the tip of the crack. Therefore, it is thought that two different behaviors, healing and damage, were observed in this condition.

**Figure 2:**
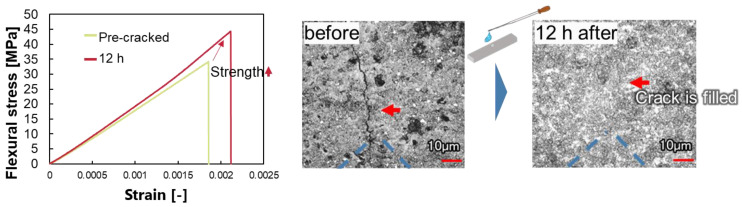
Healing behavior under pH 12.4 and [Ca^2+^] = 2.29 × 10^−4^.

Figure 2 shows the healing behavior for the case of healing with calcium hydroxide at pH 12.4. Under this condition, strength and stiffness were increased. A comparison of the cracks before and after healing showed that the cracks are completely filled under this condition. This means pH 12.4 is high enough to start dissolution and calcium ion concentration is also high enough to proceed precipitation. The product consists of fine crystals, so the product could fulfill the crack.

**Figure 3:**
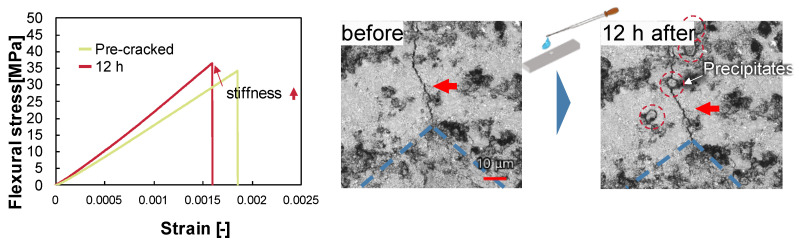
Healing behavior under pH 11.4 without Ca^2+^.

Figure 3 shows the healing behavior for the case of healing with ammonium hydroxide at pH 11.4. Under this condition, stiffness increased and strength increased only slightly. A comparison of the cracks before and after healing shows that the cracks are not filled, but some precipitates have formed on the surface. It means pH 11.4 is high enough to start dissolution and dissolved calcium ions and aluminum ions caused precipitation on the surface. The fact that crystals grow on the surface means there is a consumption of chemical components in the sample, so it is thought that the sample is damaged by the prolonged healing process. Therefore, this condition is the boundary between healing and damage. Figure 3 shows not only the concentration of chemical components but also the location of nucleation is important for healing.

**Figure 4:**
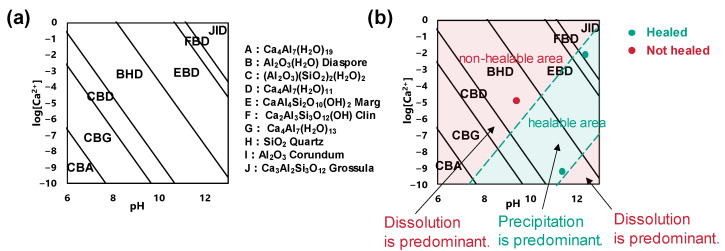
The effect of kinetics. (**a**) Chemical potential diagram (**b**) Comparison between experimental data and calculation.

To check the final product at each condition, thermodynamic calculation was performed. Calculations were performed from 15 °C to 35 °C in 5 °C increments. Only the results for 25 °C, the experimental temperature, are shown since there is no significant change in the equilibrium phase in this temperature range. Figure 4a is a chemical potential diagram that is gained by the thermodynamic calculation. Capital alphabets represent stable phases at the pH and concentration of Ca^2+^. In Figure 4b, experimental data are plotted on the diagram. Green plots are the conditions that caused an increase in strength or stiffness. The red plot is the condition that caused a decrease in strength of stiffness. Comparing the plot of pH 9.4 and the plot of pH 11.4, the healing ability is different even though their stable phase is the same. It means the possibility of healing is affected by kinetics. The point is, whether healing is possible or not is not determined by equilibrium theory alone, as has been investigated so far.

Considering the kinetics point of view, this diagram can be divided into two areas, one is a non-healable area and the other is a healable area. These regions are shown in Figure 4b. The non-healable regions are indicated by red shading, the healable regions by green shading, and their boundaries are indicated by dashed lines. The dashed lines are currently hypothetical because the boundaries are determined by experimental values and kinetics. This figure is a map of healing ability versus reaction rate (substance concentration), and it can be interpreted as follows. At relatively low pH levels, dissolution reactions predominate at low concentrations of chemical components, and subsequent gelation and crystallization reactions are slow to proceed, resulting in a non-healable region. As the pH gradually increases, the repair reaction becomes predominant along with the dissolution reaction, and the healable zone appears. If the pH concentration is high and the Ca^2+^ concentration is low, repair cannot keep up with the excessive dissolution, and a non-healable zone is expected to appear again.

**Figure 5:**
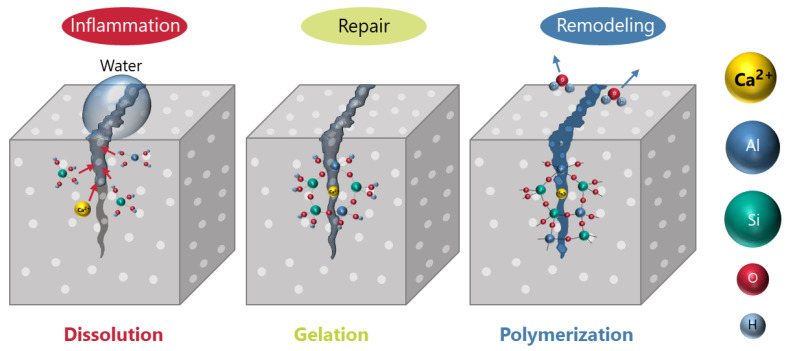
The healing mechanism of this material.

From the above, the healing mechanism can be described as follows and a schematic illustration of the mechanism is shown in Figure 5. At the inflammation stage, dissolution of chemical components like Ca, Al, and Si occur. Al and Si take the form of hydroxide. At repair stage, those hydroxides come to aggregate by the positive charge of Ca ion and to form a gel. Finally, at the remodeling stage, a polymerization reaction occurs with dehydration. We believe that controlling the kinetic competition of elementary reactions at each step is important to achieve self-healing at room temperature.

## 4. Conclusions

In this study, we assumed that the kinetic effects of elementary reaction processes must be taken into account to realize room-temperature self-healing ceramics and explored the influence of kinetic effects on the healing function. The healing behavior under different concentration conditions was examined both experimentally and computationally, and the following results were obtained.

(1) At pH 9.4, the low concentration of the chemical components prevents reactions and the transition from the inflammation stage to the repair stage does not occur easily, causing repair to fail.
(2) At pH 12.4, the concentration of chemical components is high enough to allow the transition from the inflammation stage to the repair stage, and the transition from the repair stage to the modification stage to allow recovery of strength.
(3) In the ammonium solution at pH 11.4, the reaction proceeded despite the low Ca^2+^ concentration, but strength recovery was slight. This may be because the crystals coarsened during the repair and modification stages and the precipitation reaction did not effectively work as a healing function.
(4) Comparison of the experimental results with the thermodynamic calculations indicates that the healing ability is not determined by equilibrium theory alone, but that kinetics including degradation and repair must be considered.

## Figures and Tables

**Table 1:** Chemical composition of raw materials (wt.%).

	SiO_2_	Al_2_O_3_	Fe_2_O_3_	CaO	MgO	K_2_O	Na_2_O	TiO_2_
Matrix	63.5	23.4	0.9	0.6	0.3	3.4	1.3	0.4
Cement	0.1	79.9	<0.1	18.6	0.1	-	-	<0.1

**Table 2:** Conditions of healing process.

Condition No.	pH	Temp. [°C]	Healing Time [h]	Drying Time [h]	Humidity [%]
1	9.4 (Ca(OH)_2_)	25	12	72	0
2	12.4 (Ca(OH)_2_)				
3	11.4 (NH_3_)				

## Data Availability

The datasets generated during and/or analyzed during the current study are available from the corresponding author on reasonable request.
